# Reducing the Harms of Nonclinical Psychedelics Use Through a Peer-Support Telephone Helpline

**DOI:** 10.1089/psymed.2022.0017

**Published:** 2023-06-14

**Authors:** Mollie M. Pleet, Joshua White, Joseph A. Zamaria, Rachel Yehuda

**Affiliations:** ^1^ Department of Psychiatry and Behavioral Sciences, University of California, San Francisco, San Francisco, California, USA.; ^2^ Social Neuroscience and Psychotherapy Lab, Department of Mental Health, Portland VA Research Foundation, Portland, Oregon, USA.; ^3^ Fireside Project, San Francisco, California, USA.; ^4^ James J. Peters Bronx Veterans Affairs Medical Center, Center for Psychedelic Assisted Psychotherapy and Trauma Research, Psychiatry Department, Icahn School of Medicine at Mount Sinai, New York, New York, USA.

**Keywords:** psychedelics, emergency services, harm reduction, peer support, helpline, hotline

## Abstract

**Introduction:**

A resurgence of interest in the use of psychedelics for mental health and wellness has stimulated greater experimentation with psychedelics in society. Although clinical psychedelic trials protect research participants by offering a safe setting, thorough preparation, and containment during and after ingestion of psychedelic medicines, many try these substances without the benefit of these safeguards.

**Materials and Methods:**

We analyzed data gathered from 884 callers to a psychedelic helpline to determine whether a helpline model could reduce the risks associated with nonclinical psychedelics use.

**Results:**

In total, 65.9% of callers indicated that the helpline de-escalated them from psychological distress. If not for their conversation with the helpline, 29.3% of callers indicated they may have been harmed; 12.5% indicated that they may have called 911; and 10.8% indicated they may have gone to the emergency room.

**Conclusion:**

The data suggest that access to a psychedelic helpline surrounding psychedelic experiences may avert harmful outcomes and offset the burden on emergency and medical services.

## Introduction

Evidence is emerging that psychedelic-assisted therapy (“PAT”) may be an effective treatment option for a range of psychiatric and psychological conditions. For example, psilocybin has been examined as a treatment for depression,^1–3^ and 3,4-methylenedioxy-methamphetamine (MDMA) has shown promise as a treatment for post-traumatic stress disorder.^4^ When PAT is conducted in clinical research settings, participants engage in regular medical and psychological assessments, preparation sessions with therapists, supportive dosing sessions, and postdosing “integration” sessions, during which therapists care for participants as they process the sometimes challenging psychological material that can arise during the sessions.^5^

The primary aim of this approach is to help participants derive as much meaning and therapeutic gain as possible, while also ensuring their safety. Participants in research are also protected by restrictive criteria that are designed to exclude those with contraindications such as comorbid medical or psychiatric conditions, ongoing trauma or environmental stressors, and poor social support.^6^

The safeguards that protect research participants in clinical trials of PAT are generally absent when psychedelics are consumed in nonclinical settings. This is particularly concerning, given that the use of psychedelics in nonclinical contexts is dramatically increasing. According to an NIH-funded survey released in August 2022, the use of psychedelics is at an all-time high.^7^ Although past-year psychedelics use had remained stable over the recent decades, it increased in 2021 when 8.1% of adults in the United States aged 19 to 30 years reported past-year psychedelics use (up from 5.1% in 2019 and 3.4% in 2011). Psychedelics use also increased significantly among adults aged 35 to 50 years (2.5% indicated psychedelics use in the last year, up from 0.4% in 2008).^7^

Nonclinical psychedelics use creates risks that can necessitate the use of emergency services.^8–11^ These risks are not generally associated with toxicity or overdose of the compound,^6^ but rather are psychological in nature, presenting as distress, panic, or inability to control the impact of the altered state of consciousness.^12^ In short, some people who try psychedelics nonclinically may not be prepared for the feelings of openness or vulnerability that often accompany psychedelic journeys. When there is no conduit for processing these emotions, adverse effects can occur.

In one study of 1993 psilocybin users who experienced “bad trips,” 10.7% reported that they placed themselves or others at risk of physical damage, 2.6% reported being violent or physically aggressive, and 2.7% reported having sought help in a hospital or emergency room.^8^ According to a recent survey, in 2020, 1.0% of people who consumed lysergic acid diethylamide, 1.0% who consumed MDMA, 0.6% who consumed ketamine, and 0.6% who consumed psilocybin sought emergency medical care.^9^ These percentages are concerning given that rates of psychedelics use are bound to increase, perhaps in part due to the high prevalence of mental health issues^13^ and efforts to decriminalize or legalize psychedelics.^14,15^

An absence of resources to support people using psychedelics could lead to increased reliance on an emergency medical system already overburdened by avoidable visits.^16^ Accordingly, it is imperative to address the question of how to reduce the risks associated with nonclinical psychedelics use.

In community mental health contexts, helplines such as suicide-prevention hotlines provide a useful model for how to de-escalate high-risk callers and reduce the burden on emergency services.^17,18^ For instance, in 2021 the National Suicide Prevention Lifeline, a network of crisis centers in the United States, received >3.3 million calls, chats, and texts,^19^ and crisis-line utilization is only increasing.^19^

## Materials and Methods

We sought to determine whether a helpline could reduce the risks for those experiencing difficulties during and after nonclinical psychedelics use. To do this, we conducted a pilot study using data gathered through a psychedelic helpline operated by the nonprofit organization Fireside Project. The study received ethics approval from the University of California, San Francisco Institutional Review Board (IRB no. 21-34162), which deemed participant consent unnecessary due to the anonymous nature of our data.

Fireside Project provides free peer support to people during and after their psychedelic experiences and is staffed by volunteers who undergo a 50-h training. The helpline is advertised through social media and in national and local media.

Data for this study were obtained from two sources that Fireside Project routinely uses to collect information from callers after every conversation. The first was an anonymous postcall survey (accessible at https://firesideproject.org/survey) texted to callers 24 h after every conversation. The second was call logs completed by peer-support specialists after every conversation. Each log contains questions with answer choices quoted below. Peer-support specialists were instructed to ask only questions that build rapport and address callers' needs; thus, logs do not contain responses to all questions.

## Results

Below we present data gathered between April 2021 and September 2022. During this period, surveys were sent to 4047 callers: 848 responses (20.9%) were received, and 4047 call logs were filled out by peer-support specialists.

### Postcall survey results

#### Offsetting the burden on emergency services

Of the 848 respondents, 106 callers (12.5%) indicated that if not for their conversation with a peer-support specialist, they may have called 9-1-1; 92 callers (10.8%) indicated they may have gone to the emergency room; and 249 callers (29.3%) indicated that they may have been physically or emotionally harmed.

#### De-escalating callers from distress

**Fig. 1.**
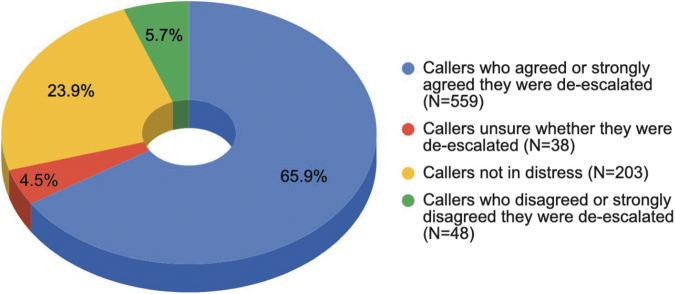
De-escalating callers in emotional, mental, or physical distress (*N* = 848).

As shown in Figure 1, helpline conversations played a significant role in de-escalating callers from emotional, mental, or physical distress.

#### Reducing risks during psychedelic integration

Of the 259 survey respondents who contacted the helpline to discuss past psychedelic experiences, 172 callers (66.4%) indicated that their conversation de-escalated them from psychological distress. If not for their conversation with a peer-support specialist, 60 callers (23.2%) indicated that they may have been physically or emotionally harmed, 16 callers (6.1%) indicated they may have called 9-1-1, and 14 callers (5.4%) indicated they would have gone to the emergency room. To our knowledge, these are the first data suggesting that a lack of support during the process of psychedelic integration may lead to harm.

### Call-log results

#### Emotional content of callers' psychedelic experiences

**Fig. 2.**
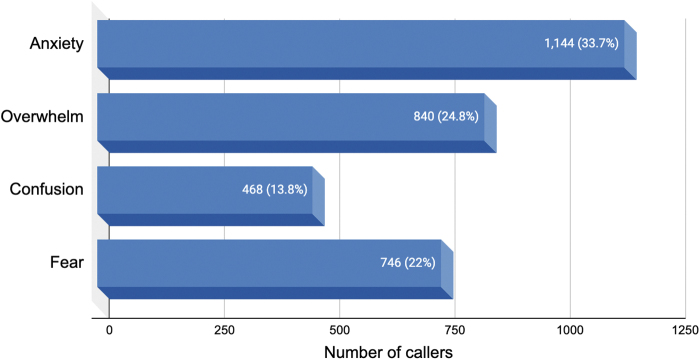
Emotional content of conversations during and after psychedelic experiences (*N* = 3386).

The call-log section entitled “Trip Content” included the following distress-specific response options: “Fear,” “Anxiety,” “Confusion,” and “Overwhelm.” Figure 2 illustrates that the 3386 callers who contacted the helpline to discuss current or past psychedelic experiences reported experiencing a range of difficult emotions.

#### Taking psychedelics alone

The call-log section entitled “Social Context” provides the following response options: “Alone,” “With one or a few close others,” “With a group of intentionally gathered people (i.e., for a ceremonial purpose),” “With a large group of known and/or unknown people,” and “Other,” with the option to enter a response. Of the 1630 call logs for callers who were in the midst of a psychedelic experience, 650 callers (39.9%) reported taking the psychedelics on their own, without other people present. Of those 650 callers, 501 callers (77.0%) were at home and 28 callers (4.3%) were outdoors in nature.

#### Consuming psychedelics with underlying psychiatric conditions

**Fig. 3.**
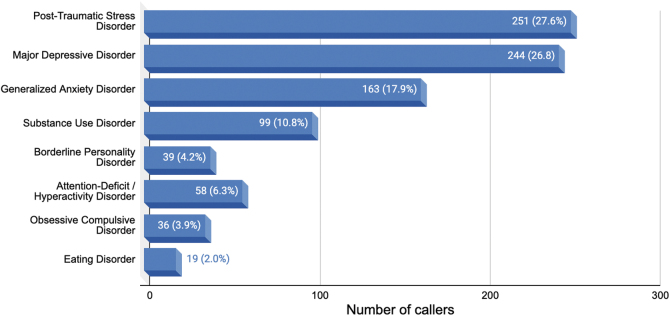
Mental health disorders mentioned by callers (*N* = 909).

Our data suggest that people may be consuming psychedelics in nonclinical contexts to address symptoms related to underlying psychiatric disorders. Of the 3386 callers who contacted Fireside to discuss current or past psychedelic experiences, 909 (27.4%) mentioned an underlying psychiatric condition. The frequency of each condition is illustrated in Figure 3.

## Discussion

Our study implies that people who use psychedelics in nonclinical settings often do so without necessary safety precautions. This creates an ethical imperative to delineate responsible solutions to reduce the risks associated with naturalistic psychedelics use. Although more research is needed, our early data suggest that access to a psychedelic helpline may avert harmful outcomes, reduce the risks sometimes associated with nonclinical psychedelics use, and offset the burden on emergency services.

### Limitations

This study has several limitations. *First*, callers are self-selecting individuals likely in need of psychedelic support. Caution should be used in generalizing results to all people who consume psychedelics in nonclinical contexts. *Second*, our survey had a 20.9% response rate. Caution should be exercised in generalizing results to all callers to the psychedelic helpline. *Third*, peer-support specialists who completed the call logs were instructed to ask only questions to build rapport and address callers' needs. As such, information in the call logs was volunteered by callers and should not be generalized to other helpline callers or to people who consume psychedelics in nonclinical contexts more generally.

*Fourth*, logs were filled out by peer-support specialists, not clinicians, which limits the validity of the question regarding psychiatric conditions. *Fifth,* although postcall survey respondents have the option to provide explanations for their answers, few actually do. This limits our ability to understand the reasons for their responses. *Finally*, because Fireside Project's call log and survey systems run on different software platforms, we were unable to link survey responses to call logs, which limited our understanding of callers' experiences. We have communicated this limitation to Fireside Project to improve data collection for future studies.
